# The Role of the CDH1 Gene in the Pathogenesis and Progression of Lobular Breast Cancer

**DOI:** 10.7759/cureus.89290

**Published:** 2025-08-03

**Authors:** Grace Mitchell, Maika G Mitchell, Preciosa Akinbo

**Affiliations:** 1 Medicine, NYU Langone Health School of Medicine, New York, USA; 2 Oncology, NYU Langone Health School of Medicine, New York, USA

**Keywords:** cdh1 gene, cell adhesion, e-cadherin, genetic mutations, lobular breast cancer, loss of heterozygosity, metastasis, targeted therapy, therapeutic resistance, tumor suppressor

## Abstract

CDH1, encoding the cell adhesion protein E-cadherin, is a critical tumor suppressor gene frequently altered in lobular breast cancer (LBC). Loss of CDH1 function, primarily through truncating or missense mutations and loss of heterozygosity, disrupts cell adhesion and polarity, promoting tumor invasiveness and metastasis. This molecular alteration defines LBC’s unique histology and clinical behavior, including distinct metastatic patterns and therapeutic resistance. While epigenetic silencing is less common, CDH1 loss correlates with poorer survival outcomes. Understanding CDH1’s role offers opportunities for targeted therapies and improved patient management, though further research is needed to address existing gaps.

## Introduction and background

CDH1 (E-cadherin) is a tumor suppressor gene that encodes a calcium-dependent cell-cell adhesion glycoprotein. Germline mutations in the CDH1 gene are strongly associated with hereditary diffuse gastric cancer (HDGC) and lobular breast cancer (LBC), a rare and invasive subtype of breast cancer. Lobular breast cancer is characterized by its lack of E-cadherin expression, which is directly linked to mutations or epigenetic silencing of the CDH1 gene. The mutation in CDH1 often leads to impaired cell adhesion and increased invasiveness, contributing to the development and progression of LBC. A significant association between CDH1 mutations and increased breast cancer risk is emphasized, particularly in hereditary cases. This stresses the importance of genetic testing in individuals with a family history of HDGC or LBC.

**Figure 1:**
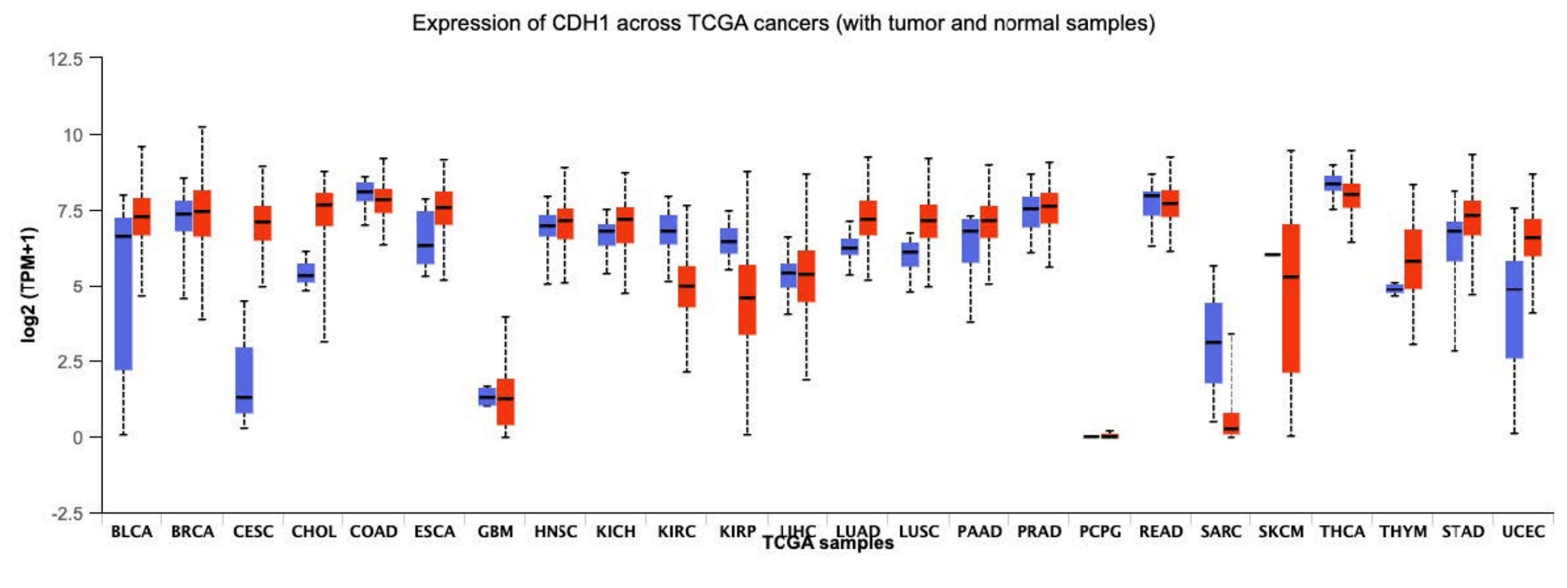
Expression of CDH1 across TCGA cancers (with tumor and normal samples). Derived from The Cancer Genome Atlas (TCGA) Breast Invasive Carcinoma (Firehose Legacy) dataset via cBioPortal [1].

**Figure 2:**
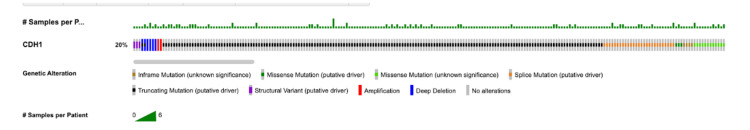
Percentage of CDH1 in Relation to Genetic Alteration. Derived from The Cancer Genome Atlas (TCGA) Breast Invasive Carcinoma (Firehose Legacy) dataset via cBioPortal [1].

As seen in Figure 1 and Figure 2, CDH1 expression is notably reduced in tumor tissues compared to normal tissues. This reduction is often due to genetic mutations or epigenetic changes like DNA methylation, which silence the gene. Mutation data for CDH1 in breast cancer reveals a prevalence of specific missense and truncating mutations, predominantly in LBC.

**Figure 3:**
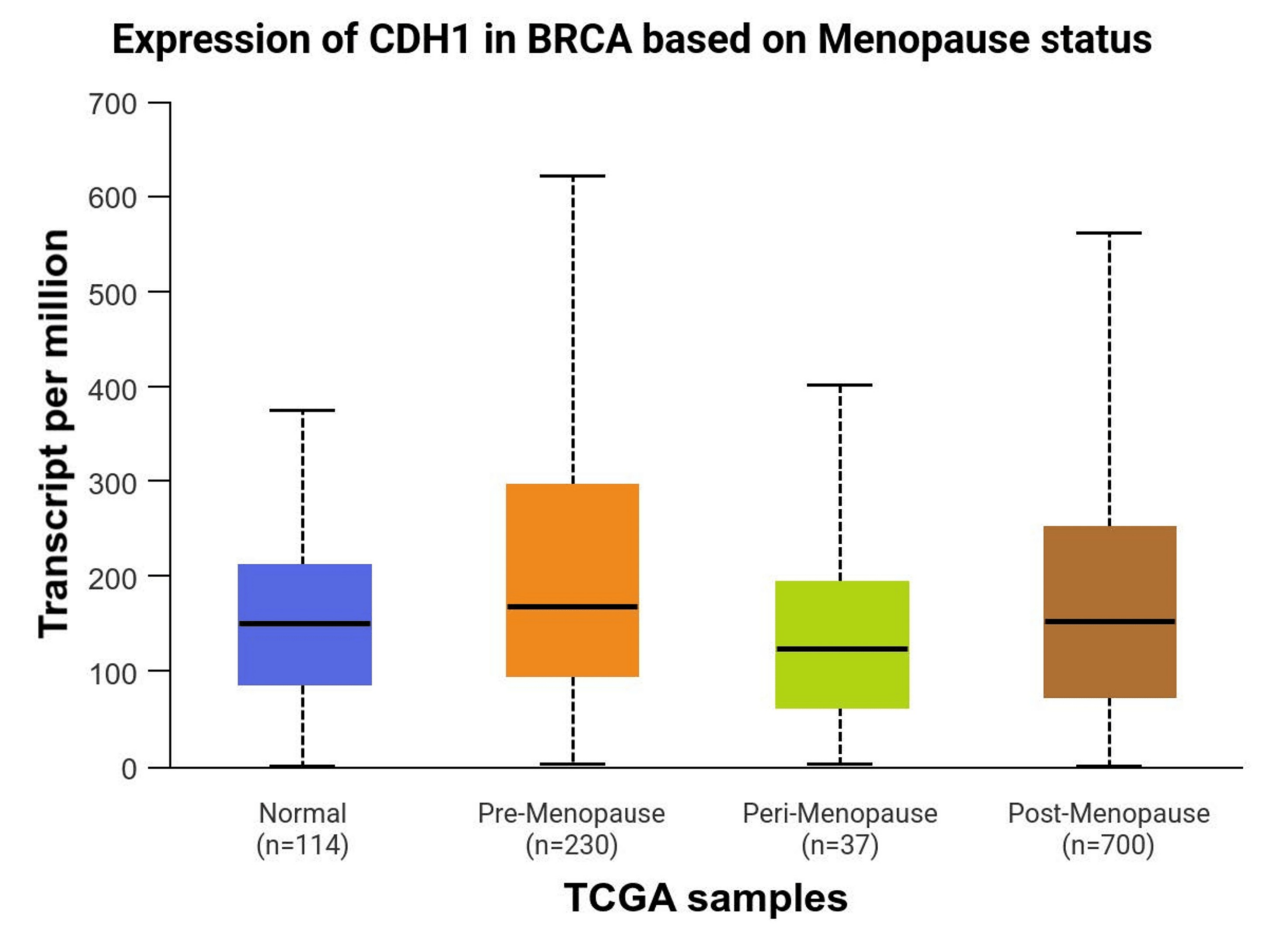
Expression of CDH1 in Breast Cancer (TCGA-BRCA) based on menopause status Data sourced from the University of Alabama at Birmingham Cancer data analysis Portal (UALCAN) [2]. “BRCA” refers to the TCGA breast cancer dataset.

**Figure 4:**
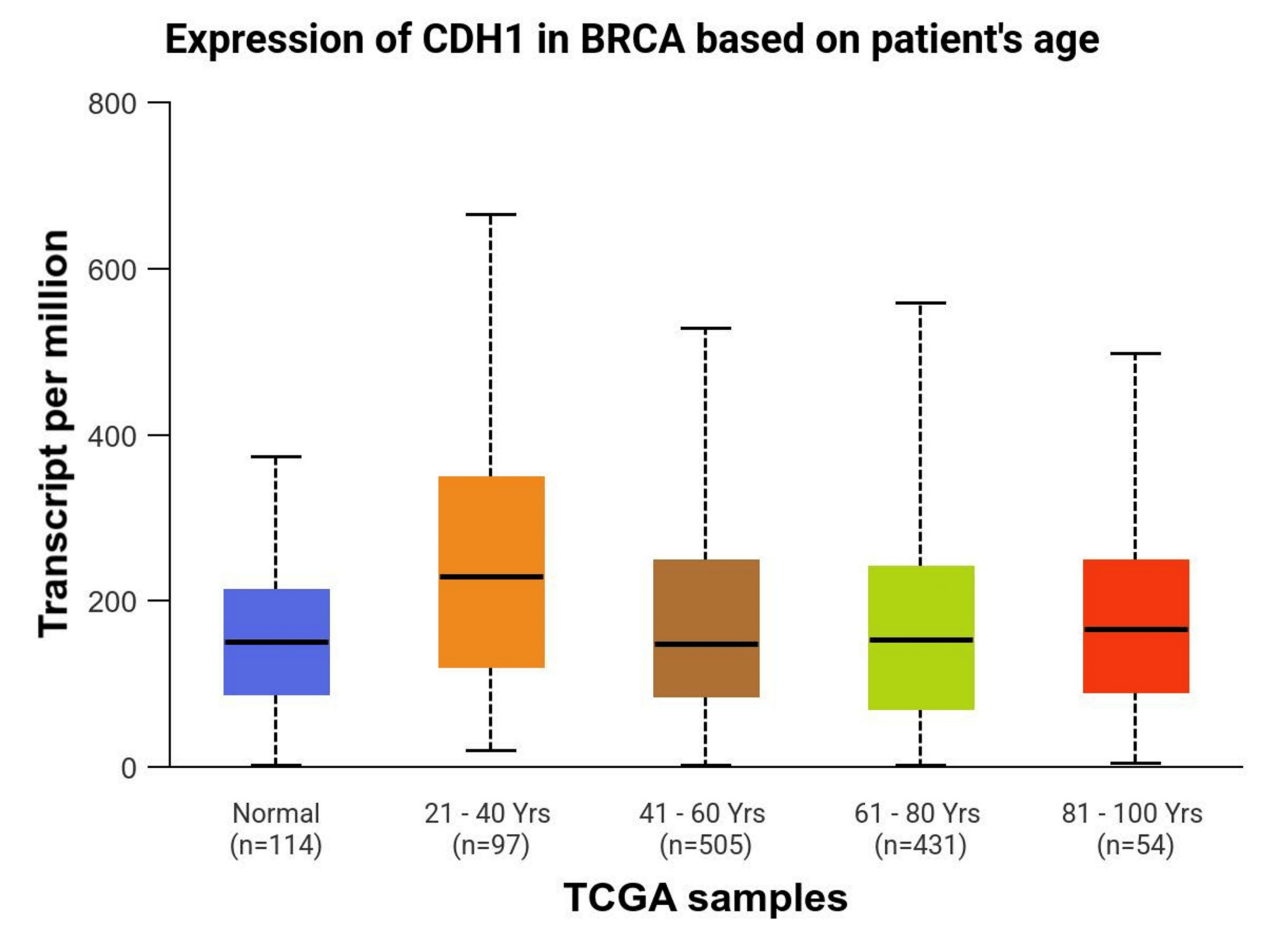
Expression of CDH1 in Breast Cancer (TCGA-BRCA) based on patient’s age. Data sourced from the University of Alabama at Birmingham Cancer data analysis Portal (UALCAN) [2]. “BRCA” refers to the TCGA breast cancer dataset.

Age and menopausal status, as can be seen in Figure 3 and Figure 4, also contribute to the expression of CDH1 in LBC. Menopausal hormonal changes could impact the expression of genes involved with cell adhesion and cancer progression. Estrogen, which is known to decrease during menopause, can contribute to the regulation of CDH1. Studies suggest that the reduction in estrogen levels during menopause may lead to alterations in CDH1 expression, potentially contributing to the development of lobular breast cancer.

**Figure 5:**
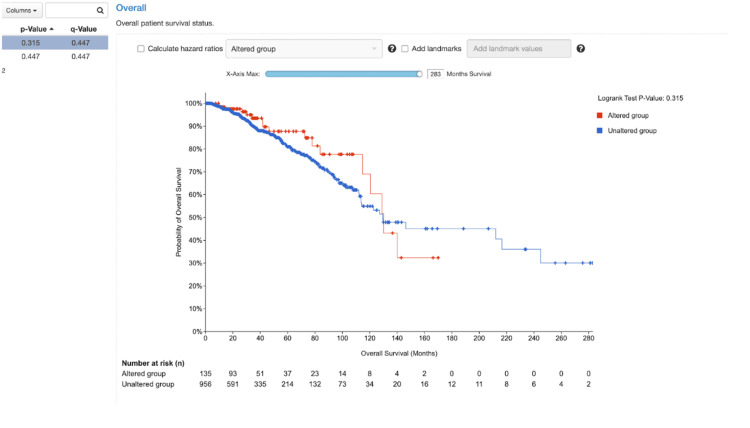
Overall patient survival status. Derived from The Cancer Genome Atlas (TCGA) Breast Invasive Carcinoma (Firehose Legacy) dataset via cBioPortal [1].

Data taken via cBioPortal can further advance the relationship between CDH1 and breast cancer, as seen in Figure 5. In Figure 5, it could be determined that as the gene expression of CDH1 decreases, the overall survival rate of breast cancer decreases. This relates to a prior connection that was made that a lack of CDH1 expression could further the progression of breast cancer.

## Review

Methods

This narrative review integrates a synthesis of peer-reviewed literature with bioinformatics analysis of publicly available datasets to explore the role of the CDH1 gene in the pathogenesis and progression of invasive lobular carcinoma (ILC) of the breast. A comprehensive literature search was conducted using PubMed, Google Scholar, and ScienceDirect to identify articles published in English up to July 2025. Search terms included combinations of keywords such as “CDH1,” “E-cadherin,” “invasive lobular carcinoma,” “lobular breast cancer,” “pathogenesis,” “gene expression,” and “tumor progression.” Boolean operators (e.g., AND, OR) were used to refine results. References of relevant articles were also screened to identify additional sources not captured in the initial search. Studies were included if they: focused on CDH1 or E-cadherin in the context of ILC, investigated molecular mechanisms, genetic alterations, or clinical implications, analyzed human-derived samples, or utilized The Cancer Genome Atlas (TCGA)-derived data. Studies were excluded if they: focused on non-breast cancers or non-ILC subtypes, examined genes other than CDH1 without relevance to E-cadherin function, were non-English, non-peer-reviewed, or editorial/opinion articles, or used animal models without translational or clinical relevance to human ILC. While a formal quantitative risk of bias tool was not applied due to the narrative nature of this review, studies were evaluated qualitatively. Preference was given to peer-reviewed articles with clear methodologies, sufficient sample sizes, and relevance to CDH1’s role in ILC. Studies with poorly defined methods, small cohorts, or ambiguous conclusions were reviewed cautiously and not emphasized in the synthesis. Findings from the literature were organized thematically into biological function, genetic alterations, expression profiles, and clinical impact of CDH1 in ILC. To complement the literature review, bioinformatics data were retrieved and analyzed from public repositories. The TCGA Breast Invasive Carcinoma (Firehose Legacy) dataset was accessed via cBioPortal [1] to examine CDH1 mutation frequency and copy number alterations in breast cancer subtypes, with a focus on invasive lobular carcinoma. Additionally, the University of Alabama at Birmingham cancer data analysis portal (UALCAN) [2] was used to evaluate CDH1 gene expression in tumor versus normal tissues within the TCGA-BRCA dataset. Expression levels were compared across subtypes when available. No new data were generated for this review; all analyses were based on de-identified, publicly available datasets.

Therapeutic strategies

Targeting CDH1-deficient tumors presents unique opportunities for personalized treatment. Due to the central role of E-cadherin in cell adhesion, its loss activates compensatory pathways that promote tumor survival and progression. One of the most commonly dysregulated pathways in CDH1-deficient LBC is the phosphoinositide 3-kinase (PI3K)/AKT signaling cascade. Studies have found that CDH1 mutations often co-occur with activating mutations in the PIK3CA gene, suggesting that PI3K inhibitors may be especially effective in this subtype [3]. Notably, data from TCGA has shown a co-enrichment of CDH1 and PIK3CA mutations in a significant proportion of LBC samples, supporting rationale for dual-pathway inhibition as a therapeutic strategy [3]. Recent studies have also identified the therapeutic potential of synthetic lethality. CDH1-deficient cells show increased sensitivity to compounds that induce oxidative stress and disrupt DNA damage repair. Boniecki et al. demonstrated that E-cadherin-deficient cells are selectively vulnerable to reactive oxygen species (ROS)-inducing agents and inhibitors of antioxidant defenses [4]. This vulnerability arises from dysregulated redox balance that makes tumor cells highly dependent on antioxidant pathways for survival. Combining ROS-inducing drugs with inhibitors of DNA repair, such as PARP inhibitors, could amplify stress and selectively eliminate CDH1-deficient cells, as has been observed in preclinical models [4]. This opens the possibility of treating LBC with agents that target redox homeostasis or DNA repair machinery. Additionally, epigenetic modulators are being explored as potential treatments. While CDH1 gene silencing through promoter methylation is not as common in LBC as initially thought, histone modifications and chromatin remodeling may contribute to gene expression changes. Drugs such as histone deacetylase inhibitors (HDACi) and DNA methyltransferase inhibitors (DNMTi) are under investigation for their ability to restore tumor suppressor expression, including CDH1 [5]. Inhibiting histone deacetylases may also reverse chromatin compaction around the CDH1 locus, allowing re-expression of E-cadherin and potentially re-sensitizing tumor cells to adhesion-mediated death pathways. Moreover, the use of DNMTi could synergize with hormonal or immune therapies by reversing epigenetically driven silencing of key immune and hormonal regulators [5]. Hormonal therapy remains a mainstay for estrogen receptor-positive LBC. Since estrogen regulates CDH1 expression, especially in premenopausal women, anti-estrogen therapies such as selective estrogen receptor modulators (SERMs) and aromatase inhibitors are commonly used [6]. However, CDH1 loss may influence responsiveness to these treatments by altering cell adhesion dynamics and signaling cross-talk. Loss of E-cadherin has been linked to ligand-independent activation of the IGF1R pathway, which may contribute to resistance to estrogen deprivation therapy. This crosstalk further underscores the need for combined targeting of hormonal and growth factor signaling in CDH1-deficient tumors [4,6]. Resistance to endocrine therapy has also been associated with co-mutations in NF1 and HER2, particularly in relapsed LBC, suggesting that HER2-targeted therapies such as neratinib may be effective in select cases [7]. Radiation therapy, particularly when combined with chemotherapy or targeted agents, has shown promise in similar subtypes of breast cancer such as triple-negative breast cancer (TNBC). A study by Khan et al. found that radiation combined with cisplatin resulted in enhanced tumor regression [8]. Given the invasive nature and poor cohesiveness of LBC tumors, radiation may also serve as an important adjunct for local control. Although LBC tumors tend to respond differently to radiotherapy compared to ductal carcinomas, the addition of radiosensitizers such as platinum compounds may help overcome intrinsic resistance mechanisms driven by E-cadherin loss and associated DNA repair deficiencies [8,9]. Recent research has also focused on combining targeted therapies with immunomodulatory agents to overcome tumor microenvironment immunosuppression in CDH1-deficient cancers. Preclinical models suggest that modulation of oxidative stress pathways may sensitize these tumors to immune checkpoint inhibitors, though clinical trials are pending [9,10]. In particular, oxidative stress appears to regulate immune recognition pathways by altering major histocompatibility complex (MHC) expression and cytokine release. Therefore, dual inhibition of redox and immune escape mechanisms may be a promising avenue for future combination regimens [4,9]. Furthermore, novel inhibitors targeting β-catenin signaling downstream of E-cadherin loss show promise in preclinical breast cancer models [11].

Mechanisms of CDH1 loss in LBC

Mutations in CDH1 are a hallmark of invasive LBC, particularly in hereditary forms. These mutations are often truncating or missense mutations that result in the loss of E-cadherin protein function. Truncating mutations, such as nonsense or frameshift alterations, prevent the full-length protein from being expressed, while missense mutations may disrupt the extracellular domain required for adhesion [12]. According to TCGA and cBioPortal data (Figure 2), CDH1 alterations are significantly more prevalent in lobular than ductal breast cancer, with the majority being truncating mutations [13]. The UALCAN portal has also demonstrated significantly reduced CDH1 mRNA expression in LBC samples compared to ductal carcinoma, supporting the link between truncating mutations and functional gene silencing [13]. Recent large-scale sequencing efforts have uncovered rarer in-frame deletions and splice site mutations that may contribute to altered E-cadherin function in LBC, highlighting the complexity of CDH1 mutational impact [14]. These less frequent alterations, although not as common as truncating mutations, can still profoundly impair protein localization and adhesion. In some cases, in-frame deletions remove key extracellular calcium-binding motifs required for adherens junction stability, further contributing to tumor cell discohesion [14]. These genetic changes directly correlate with decreased gene expression and are linked to invasive behavior and poor prognosis [3,14]. Furthermore, González-Martínez et al. demonstrated that the presence of any CDH1 gene mutation - regardless of type - was associated with reduced E-cadherin expression by immunohistochemistry, suggesting even non-truncating mutations can be functionally deleterious [14].

Although early studies proposed that CDH1 promoter hypermethylation was a frequent mechanism of silencing in LBC, more recent data challenges this assumption. Quantitative methylation-specific polymerase chain reaction (PCR) and pyrosequencing studies have shown that true promoter hypermethylation in tumor epithelial cells is rare [15,16]. Instead, prior findings were likely skewed by methylation patterns in infiltrating stromal or immune cells. Therefore, while hypermethylation may still occur in a subset of tumors, it is no longer considered a dominant mechanism of CDH1 loss in LBC. Supporting this, Caldeira et al. found that promoter hypermethylation was more commonly observed in ductal carcinoma compared to lobular subtypes, suggesting histological context influences epigenetic regulation of CDH1 [15,16]. Additionally, CDH1 silencing in LBC is more often associated with histone modifications and chromatin remodeling than promoter methylation, reinforcing the idea that alternative epigenetic mechanisms contribute more significantly to gene suppression in this context [15]. The cadherin gene superfamily, including CDH1, is known to be tightly regulated by both genetic and epigenetic inputs, and disruption at multiple regulatory levels may cooperate to reduce E-cadherin expression.

Loss of heterozygosity (LOH) at the CDH1 locus on chromosome 16q22.1 is another well-documented mechanism contributing to gene inactivation. In sporadic and hereditary LBC, LOH often accompanies a truncating mutation in the second allele, effectively resulting in biallelic inactivation [15,16]. This "two-hit" model, similar to other tumor suppressors like TP53 and BRCA1, helps explain the complete loss of E-cadherin expression in LBC tumor cells and supports the gene’s classification as a classic tumor suppressor. Figure 2 visually supports the above mechanisms by displaying the distribution of CDH1 genetic alterations from cBioPortal data [13] across breast cancer subtypes. The figure indicates that the majority of genetic events are truncating mutations (nonsense, frameshift), with lower frequencies of missense mutations and in-frame deletions. The prevalence of these alterations in LBC underscores their mechanistic relevance to the disease’s unique histology and behavior. Moreover, components of the Wnt/β-catenin pathway may be dysregulated in the context of CDH1 LOH. Loss of E-cadherin can lead to β-catenin release from the membrane, where it enters the nucleus and activates genes involved in proliferation and survival, exacerbating tumor progression [15]. This highlights how LOH at the CDH1 locus can trigger broader signaling disruptions beyond cell adhesion alone. Recent analyses by Djerroudi et al. further revealed that tumors with biallelic inactivation-whether through LOH and mutation or through two mutations-are more likely to present with high-grade histology and reduced E-cadherin staining by immunohistochemistry [11]. These findings confirm the functional consequences of LOH in CDH1-deficient LBC and support its role in defining tumor aggressiveness and progression.

Functional consequences of CDH1 inactivation

E-cadherin plays a crucial role in maintaining adherens junctions and epithelial cell polarity. Loss of CDH1 disrupts these structures, resulting in poorly cohesive tumor cells. This structural loss is what gives LBC its characteristic "single-file" growth pattern, in which cells infiltrate stromal tissue as individual units rather than cohesive clusters [16]. This pattern is not only histologically distinctive but also contributes to difficulties in early detection, as these dispersed cells are less likely to form dense masses visible on standard imaging modalities [12,15]. The absence of E-cadherin also compromises apico-basal polarity, further contributing to dysregulated tissue architecture and abnormal cell signaling. Without E-cadherin-mediated junctional complexes, epithelial cells lose spatial orientation and signaling directionality, which promotes aberrant activation of intracellular pathways like Wnt/β-catenin and PI3K/AKT [3,15]. These pathways further drive tumor cell survival and invasion, creating a feed-forward loop that reinforces tumor progression in CDH1-deficient settings. Moreover, impaired adhesion can lead to mechanical stress within the tumor microenvironment, altering extracellular matrix interactions and further disrupting polarity. This disorganization has been shown to affect not only cellular behavior but also immune surveillance and drug delivery efficiency [3,7]. Overall, the loss of adhesion and polarity plays a central role in defining the aggressive yet elusive nature of lobular breast cancer.

The lack of E-cadherin leads to enhanced invasiveness. Without cell-cell adhesion, tumor cells can more easily invade surrounding tissues and enter the lymphovascular system. Studies have shown that CDH1 loss is associated with increased metastatic potential and a higher likelihood of spread to atypical locations such as the gastrointestinal tract and peritoneum, particularly in patients with germline CDH1 mutations [12]. This pattern of metastasis reflects the discohesive phenotype of LBC cells, which enables them to infiltrate serosal surfaces and disseminate along peritoneal linings rather than forming nodular masses in the lung or liver, as commonly seen in ductal carcinomas [12,16]. Additionally, this cell dissociation contributes to difficulties in detection using traditional imaging modalities, as tumors often present diffusely. Even in the metastatic setting, lesions may appear subtle or mimic benign conditions, delaying diagnosis and treatment. This highlights the importance of incorporating clinical suspicion and molecular testing, especially in patients with known CDH1 mutations or family history of hereditary diffuse gastric or lobular breast cancer syndromes [10]. Furthermore, aberrant β-catenin signaling due to CDH1 loss can promote transcriptional activation of genes involved in extracellular matrix degradation and epithelial motility [15]. These downstream effects enable tumor cells to remodel their environment and breach tissue barriers more efficiently, enhancing their metastatic competency. The invasive capacity of CDH1-deficient tumors therefore reflects a convergence of structural, molecular, and signaling alterations that collectively promote systemic spread.

Role in epithelial-mesenchymal transition (EMT)

CDH1 inactivation is a key step in EMT, a process in which epithelial cells acquire mesenchymal traits such as motility and invasiveness. During EMT, the downregulation of E-cadherin enables β-catenin to dissociate from cell junctions and translocate to the nucleus, activating transcriptional programs associated with tumor progression [15]. Once in the nucleus, β-catenin can partner with TCF/LEF transcription factors to induce expression of EMT-related genes such as ZEB1, TWIST, and SNAI1, which further suppress epithelial markers and promote mesenchymal features [15]. This establishes a self-reinforcing loop that sustains the mesenchymal phenotype and facilitates invasion. While EMT in LBC is considered partial and context-dependent, CDH1 loss remains a major initiating factor. In particular, data from cBioPortal (Figure 5) shows a direct association between decreased CDH1 expression and reduced overall survival in breast cancer patients [13]. This supports the functional evidence that CDH1 inactivation promotes aggressive tumor behavior and contributes to worse clinical outcomes. Tumors with CDH1 alterations show lower progression-free and disease-specific survival, emphasizing the biological consequences of losing E-cadherin. Moreover, EMT-like changes in LBC may be more subtle than in other cancers, occurring without complete loss of epithelial markers. This "hybrid" state may allow tumor cells to retain some adhesive properties while still gaining invasive potential, making them harder to target therapeutically and more adaptable during metastasis [16].

CDH1 and clinical outcomes: prognostic value of CDH1 loss

Loss of CDH1 expression is a defining feature of invasive LBC and has notable prognostic implications. While LBC is typically estrogen receptor-positive and historically considered to have a favorable prognosis, emerging molecular evidence suggests that CDH1 loss contributes to distinct clinical behavior. Tumors with CDH1 alterations often present at larger sizes, have higher rates of nodal involvement, and show late distant metastases, particularly to the gastrointestinal tract, peritoneum, and ovaries [12]. These metastatic tendencies are not random; rather, they reflect the biological consequences of impaired cell adhesion and altered tissue tropism driven by CDH1 inactivation. These unusual metastatic patterns are believed to stem from the non-cohesive architecture resulting from E-cadherin loss. Additionally, CDH1 loss has been associated with lobular histology that is less responsive to traditional endocrine therapies over time, especially when concurrent mutations (e.g., in HER2 or NF1) are present [7]. In particular, studies have observed that CDH1-deficient tumors often exhibit molecular profiles characterized by PI3K pathway activation and immune evasion, which may blunt responsiveness to both hormonal and immunomodulatory treatments [3,5]. This suggests that CDH1 status could serve as a predictive biomarker not only for prognosis but also for therapeutic responsiveness, especially in the context of emerging targeted and immune-based therapies. Moreover, the pattern of delayed recurrence in LBC highlights the need for extended follow-up in CDH1-mutated cases. These tumors may remain clinically dormant before re-emerging in unusual anatomical sites, making recurrence patterns harder to detect without vigilant long-term monitoring [12]. Taken together, CDH1 inactivation shapes not only the morphology of LBC but also its clinical trajectory, with implications for diagnosis, treatment planning, and surveillance strategy.

Survival rates, disease progression, and therapeutic resistance

Several studies have demonstrated that patients with CDH1-mutated LBC have worse long-term survival outcomes compared to patients with ductal carcinoma or lobular tumors without CDH1 alterations. In particular, disease-specific survival is negatively impacted in tumors with biallelic CDH1 inactivation [3]. This is consistent with the loss of both adhesion and polarity, which facilitates diffuse invasion and late-stage detection, ultimately worsening prognosis. Tumors with concurrent alterations in CDH1 and PIK3CA or PTEN show particularly aggressive behavior and may require combination therapy to overcome resistance mechanisms [3]. These tumors may also exhibit resistance to endocrine therapy, especially aromatase inhibitors, due to downstream signaling alterations. Additionally, patients with hereditary LBC due to germline CDH1 mutations may develop multifocal or bilateral disease, which complicates treatment and surveillance strategies [6]. Prophylactic mastectomy is sometimes considered in these patients, similar to management approaches in BRCA1/2 mutation carriers, due to the high risk of occult or multifocal disease at diagnosis [12]. As shown in Figure 5, survival data obtained through cBioPortal [13] supports a direct relationship between decreased CDH1 expression and poor overall survival. Patients with CDH1-deficient tumors had significantly shorter survival times, particularly in the absence of co-expression of adhesion-related genes. This data reinforces the clinical impact of CDH1 loss, not only as a diagnostic marker but as a prognostic indicator. The inclusion of CDH1 mutation status in molecular diagnostic panels could help guide treatment selection and surveillance intensity. Furthermore, resistance to treatment in CDH1-deficient tumors may be mediated by compensatory upregulation of growth factor receptors, such as IGF1R or HER2, which provide alternate survival pathways in the absence of E-cadherin-mediated signaling [4,7]. Targeting these co-activated pathways may improve outcomes in select cases of refractory LBC.

Future directions and research gaps

Despite progress in understanding the molecular role of CDH1 in lobular breast cancer, several gaps in research remain. One critical area involves refining the methods used to detect CDH1 silencing. Early studies reporting high frequencies of CDH1 methylation were based on non-quantitative methods, and more recent research suggests that true promoter methylation in tumor cells is rare [13]. Accurate detection methods that distinguish tumor-specific from stromal methylation are needed to clarify the role of epigenetics in CDH1 loss. Emerging technologies such as single-cell methylation sequencing and digital droplet PCR could provide the resolution needed to dissect epigenetic heterogeneity within tumor microenvironments, thereby refining biomarker accuracy [13,14]. Another important area involves identifying co-altered pathways and cooperating mutations. While CDH1 loss alone contributes to the lobular phenotype, other mutations such as those in PIK3CA, PTEN, or TP53 are often co-occurring and may influence prognosis and treatment response [1]. Comprehensive molecular profiling of these co-mutations could uncover new vulnerabilities and improve treatment personalization. Multi-omics integration, combining genomics, transcriptomics, and epigenomics, holds promise to map the complex interactions driving LBC progression and therapy resistance, as suggested by data from cBioPortal and TCGA [10,11]. Immunotherapy response in LBC is another largely unexplored field. While LBC tumors are generally considered “cold” due to low tumor mutational burden and limited immune infiltration, recent evidence suggests that CDH1 loss may alter immune cell composition within the tumor microenvironment [5]. Studies are needed to evaluate whether CDH1-deficient LBCs could benefit from immune checkpoint blockade, especially when combined with treatments that increase immunogenicity. Preclinical data indicate that targeting oxidative stress pathways may sensitize tumors to immunotherapy by enhancing antigen presentation and reversing immune evasion, but clinical trials specifically focused on CDH1-mutant LBC are lacking [9]. Clinical trials specific to LBC or CDH1-mutant breast cancer are limited. Most large-scale breast cancer trials either exclude LBC or do not stratify patients based on histological or molecular subtype. As CDH1 loss becomes more recognized for its biological and clinical impact, future trials should incorporate this molecular stratification into their design. Biomarker-driven enrollment could improve the development of CDH1-targeted therapies and enhance clinical outcomes. Furthermore, liquid biopsy approaches leveraging circulating tumor DNA to detect CDH1 mutations non-invasively may facilitate early detection of recurrence and real-time monitoring of treatment response [10]. Moreover, advances in liquid biopsy techniques may allow non-invasive detection of CDH1 mutations and monitoring of treatment response in LBC patients, offering a valuable tool for personalized management [10]. Integration of multi-omics data including genomics, transcriptomics, and epigenomics is likely to deepen our understanding of CDH1-related tumor biology and identify novel targets [11]. Such integrative analyses could also uncover previously unrecognized regulatory networks or feedback loops involving E-cadherin and its downstream effectors, opening new therapeutic avenues. Continued research efforts are essential to translate molecular insights into improved clinical management for patients with LBC.

## Conclusions

Ultimately, CDH1 loss plays a central role in the development and progression of lobular breast cancer. Through a combination of genetic mutations, loss of heterozygosity, and, less frequently, epigenetic silencing, E-cadherin function is disrupted, leading to a distinct histologic subtype with unique clinical features. Functionally, CDH1 loss impairs cell adhesion, promotes invasion, and activates partial epithelial-mesenchymal transition. Clinically, this is associated with altered patterns of metastasis, therapeutic resistance, and poorer long-term survival. While targeted therapies and new treatment strategies are being developed to exploit CDH1-associated vulnerabilities, significant research gaps remain. Expanding molecular testing, improving detection methods, and designing LBC-specific clinical trials are critical next steps. Understanding and addressing CDH1 loss at every level-from molecular biology to clinical management-has the potential to greatly improve outcomes for patients with this under-recognized subtype of breast cancer.

## References

[1] (2025). TCGA Breast Invasive Carcinoma (Firehose Legacy) [dataset]. cBioPortal for Cancer Genomics. https://www.cbioportal.org/study/summary.

[2] UALCAN UALCAN (2025). TCGA gene analysis: CDH1 expression in BRCA. https://ualcan.path.uab.edu/cgi-bin/TCGA-survival1.pl.

[3] Ciriello G, Gatza ML, Beck AH (2015). Comprehensive molecular portraits of invasive lobular breast cancer. Cell.

[4] Elangovan A, Hooda J, Savariau L (2022). Loss of E-cadherin induces IGF1R activation and reveals a targetable pathway in invasive lobular breast carcinoma. Mol Cancer Res.

[5] Connolly R, Stearns V (2012). Epigenetics as a therapeutic target in breast cancer. J Mammary Gland Biol Neoplasia.

[6] Mangani S, Piperigkou Z, Koletsis NE, Ioannou P, Karamanos NK (2025). Estrogen receptors and extracellular matrix: the critical interplay in cancer development and progression. FEBS J.

[7] Ross JS, Wang K, Sheehan CE (2013). Relapsed classic E-cadherin (CDH1)-mutated invasive lobular breast cancer shows a high frequency of HER2 (ERBB2) gene mutations. Clin Cancer Res.

[8] Khan MS, Khan MA, Gabbidon K (2021). The combination of radiation and cisplatin enhances tumor regression in triple-negative breast cancer. Cancers (Basel).

[9] Kaelin WG Jr (2017). The concept of synthetic lethality in the context of anticancer therapy. Nat Rev Cancer.

[10] Djerroudi L, Bendali A, Fuhrmann L (2024). E-cadherin mutational landscape and outcomes in breast invasive lobular carcinoma. Mod Pathol.

[11] Corso G, Intra M, Trentin C, Veronesi P, Galimberti V (2016). CDH1 germline mutations and hereditary lobular breast cancer. Fam Cancer.

[12] González-Martínez S, Kajabova VH, Pérez-Mies B (2024). CDH1 methylation analysis in invasive lobular breast carcinomas with and without gene mutation. Virchows Arch.

[13] Caldeira JR, Prando EC, Quevedo FC, Neto FA, Rainho CA, Rogatto SR (2006). CDH1 promoter hypermethylation and E-cadherin protein expression in infiltrating breast cancer. BMC Cancer.

[14] Bouras E, Karakioulaki M, Bougioukas KI, Aivaliotis M, Tzimagiorgis G, Chourdakis M (2019). Gene promoter methylation and cancer: an umbrella review. Gene.

[15] Berx G, van Roy F (2009). Involvement of members of the cadherin superfamily in cancer. Cold Spring Harb Perspect Biol.

[16] Song P, Gao Z, Bao Y (2024). Wnt/β-catenin signaling pathway in carcinogenesis and cancer therapy. J Hematol Oncol.

